# Infectivity of* Mycobacterium tuberculosis* Genotypes and Outcome of Contact Investigation in Classroom in Guangxi, China

**DOI:** 10.1155/2019/3980658

**Published:** 2019-04-14

**Authors:** Dongxiang Pan, Prasit Palittapongarnpim, Angkana Chaiprasert, Mei Lin, Dingwen Lin, Xi Long, Liwen Huang, Huifang Qin, Edward McNeil, Rushu Lan, Xiaoqiang Qiu, Virasakdi Chongsuvivatwong

**Affiliations:** ^1^School of Public Health, Guangxi Medical University, Nanning, Guangxi, 530021, China; ^2^Department of Microbiology, Faculty of Science, Mahidol University, Bangkok, 10700, Thailand; ^3^National Science and Technology Development Agency, Pathum Thani, 12120, Thailand; ^4^Office of Research and Development, Faculty of Medicine Siriraj Hospital, Mahidol University, Bangkok, 10700, Thailand; ^5^Department of Tuberculosis Prevention and Control, Guangxi Zhuang Autonomous Region Center for Disease Prevention and Control, Nanning, Guangxi, 530021, China; ^6^Epidemiology Unit, Faculty of Medicine, Prince of Songkla University, Hatyai, Songkhla, 90110, Thailand

## Abstract

**Objective:**

To evaluate the infectivity of* Mycobacterium tuberculosis* (*M.tb*) genotypes of index cases in the classroom of adolescent schools in Guangxi, China.

**Methods:**

Adolescent school tuberculosis (TB) contact investigations were conducted for all reported index TB cases from November 2016 to December 2017 in Guangxi, China. Genotypes of index cases and contact cases were identified by 15-loci mycobacterial interspersed repetitive units–variable number tandem repeat and spoligotyping. Outcome variable was 5 levels' order of tuberculin skin test (TST) results to new active TB [0-5 mm, 6-9 mm, 10-14 mm, ≥ 15 mm (without TB), and ≥15 mm (with TB)]. Multivariate ordered logistic regression analysis was performed to evaluate the independent effect of genotypes of index case on contact screening outcome.

**Results:**

Beijing genotype occurred more commonly in female index patients. One genotypic cluster of two index cases and one cluster of two contact cases were detected. The association between infectivity of Beijing genotype of index cases and outcome of contact investigation was statistically significant in univariate analysis but no so after adjustment for characteristics of contacts and sex of index cases (*P value*=0.057). Female index cases increased the chance for TB infection/being active TB among contacts (ordinal odds ratio = 1.39, 95% confidence interval: 1.21, 1.60). Contacts who studied in the middle school, who with non-Han ethnicity and who without BCG scar had increased risk for TB infection/being active TB.

**Conclusion:**

There was not enough evidence from our data to support that Beijing strains were more infective than non-Beijing strains in TB transmission in school setting.

## 1. Introduction

Tuberculosis (TB) is an infectious disease caused by the bacillus* Mycobacterium tuberculosis (M.tb)*. It was estimated that approximately 1.7 billion people (approximately one-quarter of the world's population) were latently infected with* M.tb* globally [1]. Prevention of new infections of* M.tb* and their progression to TB disease is critical to reduce the burden of disease and death caused by TB and to achieve the End TB Strategy targets set for 2030 and 2035 [2].

Contact tracing plays a key role in identifying new TB cases and interrupting* M.tb* transmission [3]. The Beijing genotype family was well recognized as a distinct genetic lineage and the major concern of* M.tb* [4]. It had a worldwide distribution but predominates in certain geographic areas, particularly in parts of Asia [5]. Variable frequency of Beijing genotype was illustrated from previous studies, such as 86% in China [6], 50% in Mongolia [7], 43% in South Korea [7], 44% in Thailand [8], 53% in Vietnam [9], 34% in Indonesia [10], and 25% in Malaysia [11]. Compared to other genotypes, Beijing genotype family reported association with TB outbreaks, enhanced pathogenicity, and a predilection for drug resistance [4, 12, 13]. The ubiquity of the Beijing strains and their frequent association with outbreaks and drug resistance emphasize their importance [4].

However, the mechanisms underlying the spread and pathogenesis of TB disease of Beijing family and other strains in the human population are not well documented. The association of* M.tb* strain infection with epidemiological manifestations is not systematic, especially in the contact investigation setting. Therefore, it is necessary to further explore the infectivity of* M.tb* genotypes and related epidemiological linkage to better understand the nature of* M.tb* strains and interrupt the transmission.

In China, when school students with newly reported TB (“index cases”) are identified, school contact investigation is conducted for index cases' classmates and teachers (“contacts”) and then to determine whether contacts have TB infection or active TB. In this study, we aimed to evaluate genotypes of* M. tb* of the index cases and the outcome of contact investigation and to test the assumption whether the Beijing family of the index case was more infectious to school contacts than non-Beijing genotypes after adjustment with various epidemiological factors, such as school type, sex, ethnicity, and clinical characteristics.

## 2. Methods

### 2.1. Study Design

School-based tuberculosis contact investigations and molecular genotyping of* Mycobacterium tuberculosis *were conducted in Guangxi Zhuang Autonomous Region (Guangxi), which has a population of 50 million and is one of the highest TB burden provinces in China.

### 2.2. Study Subjects

Study subjects were index TB cases notified to Guangxi CDC during November 2016 to December 2017 and classroom contacts of the index TB cases.

### 2.3. Identification of Contacts

Contacts of the index TB case were identified according to the national guideline [14]. The classroom contact was referred to those who had direct contact with the index case in the same class, including classmates and teachers.

### 2.4. Contact Screening

#### 2.4.1. Adolescent Index TB Case Identification

New active pulmonary TB cases were diagnosed at the county CDC clinics or hospitals and were immediately notified to the Guangxi CDC, where the study team led by the first author undertook the contact investigation within 15 days. An active pulmonary TB case was defined according to the diagnosis criteria recommended by WHO [15]. In more detail, a case could be confirmed by sputum smear and/or culture for* M.tb* or only clinically diagnosed based on chest radiography without bacteriological conformation.

Index cases were school students who studied in a middle school or a high school and were diagnosed with new active pulmonary TB case.

### 2.5. Contact Investigation

Investigation of contacts included tuberculin skin test (TST) and questionnaire. Detail of contact investigation has been described in previous study [16]. Briefly, the investigated procedures included the following:

(1) Interview questionnaire. It included information on age, gender, ethnicity, school type, birth place, BCG scar, suspected symptoms of TB, and other pertinent medical history.

(2) Tuberculin skin test (TST). Because of the high BCG vaccination rate, Chinese guidelines recommend that students with an induration diameter ≥ 15 mm be defined as TST positive [17].

(3) Chest radiography and sputum examination. All students with a positive TST or who had suspected TB symptoms had a chest radiograph performed and sputum examination. Further diagnostic procedures for active TB followed the diagnostic criteria by WHO recommendation [15].

### 2.6. Molecular Genotyping of* Mycobacterium tuberculosis*

Sputum smear culture was performed for index cases and contacts TB cases. Isolates were cultured on Löwenstein-Jensen medium slants (Becton, Dickinson, and Co., Franklin Lakes, NJ) for 3 to 4 weeks at 37°C under 5% CO_2_. The isolated bacteria were processed for identification, drug susceptibility testing, 15-loci mycobacterial interspersed repetitive units–variable number tandem repeat (MIRU–VNTR) typing, and spoligotyping.

Susceptibility to rifampicin, isoniazid, ethambutol, streptomycin, amikacin, capreomycin, and ofloxacin was determined by the indirect absolute concentration method [18].

MIRU-VNTR genotyping was performed by PCR amplification of 15 loci (Mtub04, ETRC, MIRU04, MIRU40, MIRU10, MIRU16, Mtub21, QUB11b, ETRA, Mtub30, MIRU26, ETRE, Mtub39, QUB26, and QUB415) according to the manufacturer's protocol. MIRU-VNTR profiles obtained from the strains tested were identified in the MIRU-VNTR plus database [19].

Spoligotyping was using one-step spoligotyping protocol termed McSpoligotyping based on real-time PCR, which was described by Xiaohong Zeng et al.[20]. McSpoligotyping could be used in epidemiology studies of tuberculosis by taking advantage of the shortened procedure, ease of use, and compatible results with standard spoligotyping [20]. Spoligotyping results were converted into octal codes and entered in SITVIT database for analysis.

Molecular clustering and Beijing/non-Beijing family genotypes of the isolates were determined by constructing a dendrogram based on MIRU-VNTR and spoligotyping data. A cluster was defined as two or more isolates sharing completely identical fingerprints based on both MIRU-VNTR and spoligotyping methods. A phylogenetic tree was constructed using the software program MEGA7 [21].

### 2.7. Outcome Variable

The primary outcome was contact screening outcome [including TST positive (induration diameter≥ 15 mm) and being new active TB]. The contact screening outcome was divided into 5 ordinal levels [contacts with induration diameter of 0-5 mm, 6-9 mm, 10-14 mm, ≥ 15 mm (without active TB diseases), and ≥ 15 mm (with pulmonary TB)].

### 2.8. Statistical Analysis

There were two levels of unit of analysis. The first level was index case unit, through comparison of background characteristics and clinical characteristic of the index cases with genotypes [Beijing genotype, non-Beijing genotypes, and unknown genotype (those index cases were culture negative or did not have isolates)]. The second level was contacts of the index cases. Comparison between contacts background characteristic and contact screening outcome with genotypes of index cases were evaluated. As the outcome variable was an ordinal variable, multivariate ordered logistic regression analysis was performed to identify the effect of genotypes of index case to outcome of contact screening after adjustment for potential confounders. The models were used incrementally to include potential confounders such as sex, ethnicity, and presence of BCG scar to check whether the independent effect was significant when all confounders were in place. The model with the minimum Akaike Information Criterion (AIC) was used to choose the best model [22]. The final significance level for regression analyses was set at <0.05.

## 3. Results

A total of 6512 contacts of 117 index TB cases classes were investigated. Of the 117 index classes, 105 from 105 individual schools, and four sets of 2 classes were from 4 schools and 1 set of 3 classes were from 1 school. The mean ± standard deviation age of index cases was 16.7 ± 1.9 years, and 45.3% (53/117) were male. Sputum positive acid-fast bacillus (AFB) smears were reported in 35.9% (42/117) of the indexed cases.

### 3.1. New Active TB Cases among Contacts of Index Cases

Of 6512 contacts that were screened, 223 (3.42%) were TST positive and underwent chest radiography and sputum examination, and 45 were diagnosed as new active TB (9 with culture positive sputum). The number needed for screening to find one new TB case was 145 (6512/45) (95% CI: 107.27, 197.23). There were 151 teachers who were screened and found to be negative for active TB. The teacher population was not further analyzed.

### 3.2. TST Results and New Active TB Detection of Contact Screening

**Figure 1:**
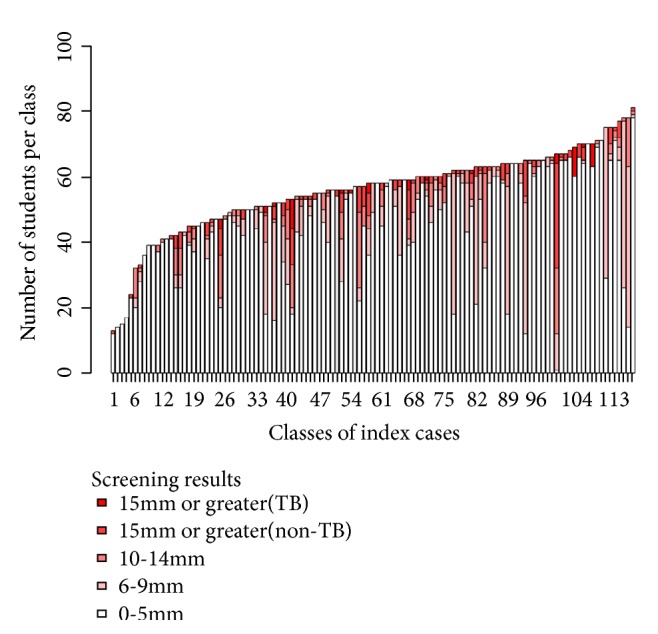
TST screening result distribution for each contact class sorted by the class size.

Figure 1 summarizes the distribution of contact screening outcome (TST results, including new active TB detection) in each index case class sorted by class size (number of students per class). The height of each pillar denoted total number of students in each class. Different shades indicated the level of screening results (TST results and contact TB case) from 0-5 mm, 6-9 mm, 10-14 mm, ≥ 15 mm (without active TB diseases), and ≥ 15 mm (with active pulmonary TB). There is no obvious relationship between class size and distribution of TST results. However, cut-off with a number of students more than 52 students per class group increased 2.45 (95% CI: 1.01, 6.02) times risk of detection with TST positive compared to classroom with less than or equal to 52 students' group.

### 3.3. Spoligotyping and 15-Loci MIRU-VNTR Genotyping Results

A total of 23 isolates were collected, including 3* non-Tuberculous mycobacteria *(one was index case and two were contacts TB cases) isolates and 20* M.tb *isolates. The latter were from 18 schools and comprised 16 index case strains and 4 contacts strains. All the strains were fully drug-sensitive.

**Figure 2:**
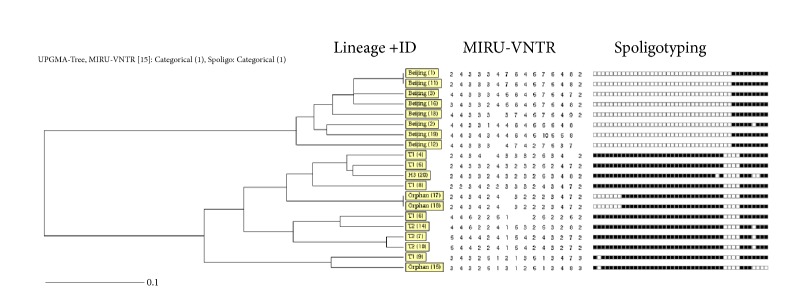
15-loci MIRU-VNTR and spoligotyping pattern of index cases and contact cases.

Figure 2 showed 15-loci MIRU-VNTR and spoligotyping pattern of index cases and contact cases with* M.tb *isolates. Using spoligotyping method, the isolates were classified into 4 spoligotypic clades and 3 orphan types, comprising eight Beijing isolates, five T1 isolates, three T2 isolates, and one H3 isolate. All the non-Beijing isolates belonged to the Euro-American Family.

**Figure 3:**
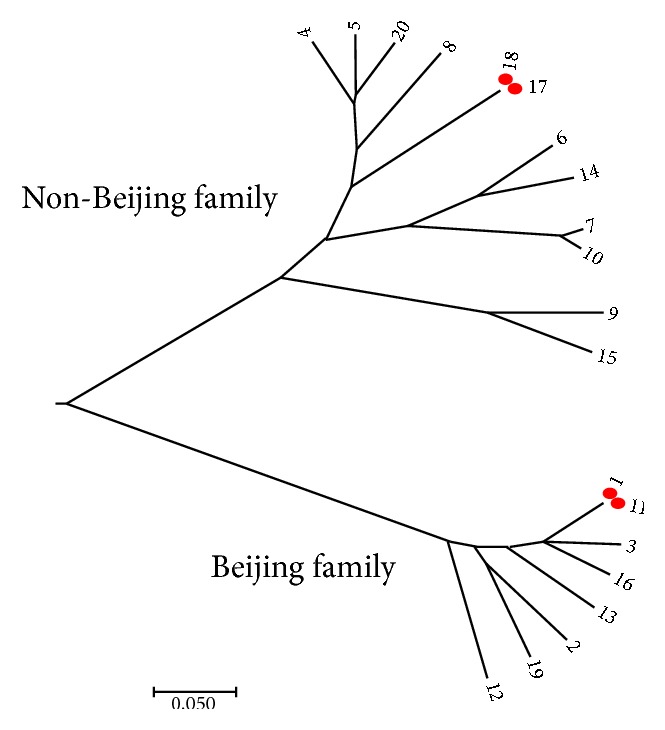
The phylogenetic tree of 16 index case isolates and 4 contacts isolates. Note: the number represented the isolate ID number. ID numbers 1-16 were index cases isolates; ID numbers 17-20 were contact cases isolates.

Figure 3 showed the phylogenetic tree of VNTR genotypes of 20* M.tb *isolates, with 4 isolates being clustered in two clusters. The genotypes of the other 16 isolates were unique. The cluster of the non-Beijing isolates that comprised 2 isolates was from two classmates of the same index case (did not have index case genotype), and the cluster of the Beijing family was from two index cases in different schools. Both latter subjects had the same birth place but no other social connections. We did not find a cluster of the index case and contact cases.

### 3.4. Characteristics of Index Case by Genotype

Table 1 summarizes the relationship of characteristics and genotypes of index cases. There was a statistically significant association between the sex of the index cases and genotypes. The other characteristics, such as school type, ethnicity, and clinical characteristics (BCG scar, sputum smear status, and cavity on chest) did not have statistically significant association with genotypes of index case.

### 3.5. Associations between Characteristics of Contacts and Outcome of the Contact Screening by Genotypes of Index Cases

Table 2 summarizes the relationship of characteristics of contacts and contact screening outcomes (TST results and new active TB detection) by genotype of index cases. Characteristics of contact, school types, sex, ethnicity and BCG scar, had statistically significant association with genotypes of index cases.

### 3.6. Ordered Logistic Regression Model Assessed Contacts Screening Outcome by the Genotypes of Index Case

Since genotypes of index cases were associated with sex of index case and characteristics of contacts, school types, sex, ethnicity, and BCG scar, as well as contact screening outcome, we ran ordered logistic regression predicting the contact screening outcome (TST positive/ being new TB), having above variables as independent variables.

Table 3 summarizes models with covariates incrementally added. Model 6 had the lowest AIC value, so this model was chosen as the best model.

Table 4 provides the effect of genotypes of index cases to the outcome of contact screening (TST positive/being new TB) in model 6. Using the non-Beijing genotype of index case as a reference, Beijing genotype of index case was not significantly associated with contact screening outcome (ordinal adjusted OR = 1.34, 95% CI: 0.93, 1.95). Having female index cases, contacts studied in the middle school, with non-Han ethnicity and absence of BCG scar, were identified as the high-risk group for positive outcome of screening.

**Figure 4:**
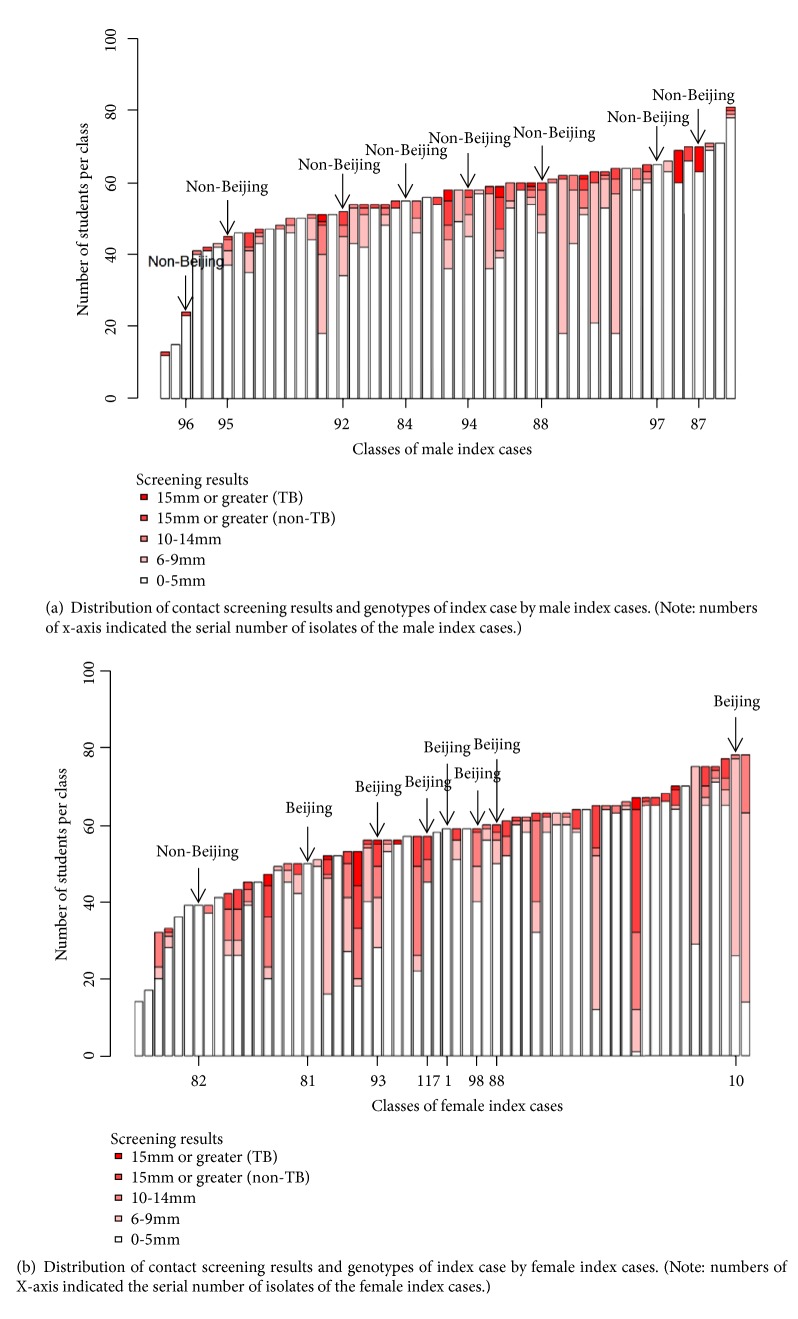


Figures 4(a) and 4(b) summarize the distribution of contact screening outcome and genotype of index cases by sex of index cases. All of male index cases were seen with non-Beijing genotype (8/8, Figure 4(a)) and most of female index cases were seen with Beijing genotype (7/8, Figure 4(b)).

## 4. Discussion

There was a strong association between Beijing genotype and female of index patients as well as contacts characteristics in our study. Risk factors for increasing positive outcome of contact screening included female index cases, contacts who studied in the middle school, non-Han ethnicity, and absence of BCG scar. The association between the Beijing genotype of index case and the contact screening outcome (TST positive/being new TB) was not statistically significant after adjustment of the potential confounders.

Beijing strains were suggested to have a selective advantage over other* M.tb *lineages as conferred through increased transmissibility and virulence system [23–25]. It might be with greater virulence as a result of intrinsic biochemical properties and their interaction with human host defense system [23]. In previous studies, Beijing strains were presented more likely than non-Beijing strains to be in a genotypic cluster, had higher rate of progression to active TB, or associated with drug resistance [26–28]. However, we did not find significant association between infectivity of Beijing genotypes of index cases and contact investigation outcome, and the Beijing genotype was unrelated to drug resistance. This was consistent with previous studies conducted in low TB incidence settings such as in the United States [29], Netherlands [30], and Canada [31], which demonstrated that transmission of Beijing strains posed no more of a public health threat than non-Beijing strains. The association with increased transmission of Beijing strains in some settings may reflect geographic variations in virulence phenotypes, difference in TB prevalence, and the population with drug resistance. It was also suggested that modern lineages (sublineages of Beijing family) induced weaker immune responses than ancient lineages (sublineages of Beijing family) did, and this response possibly provides modern lineages with a selective advantage in terms of more rapid disease progression and transmission in the human population [32]. To better understand the potential implications of virulence system, future population-based investigations in high- and low-incidence settings discerned between the disease characteristics and transmissibility of different* M. tb* subfamilies or sublineages are needed.

Our findings showed that female index cases increased 1.39 times of the TST positive more than the male index cases did. The possible explanations for the difference could be the following. (1) The contact pattern of female cases might present more closeness to the contacts than male cases [33]. It was well documented that contact history and closeness of contact with index case were risk factors for TB disease [34, 35]. Moreover, females were more likely to stay indoors such as classroom or dorm room than outside, where a corresponding higher infectious dose could contribute to spread of the disease. (2) The fear and stigma associated with TB seem to have a greater impact on female than on male [36]. Female students might be more worried that the disease would affect the study and then ignore or hide the disease, which resulted in unnecessary transmission in school.

Absence of BCG scars was found to be an independent factor associated with positive result of contact investigation, which was consistent with studies in Turkey [37], India [38], and rural China [39]. School type was also associated with the outcome of contact investigation. One reasonable explanation might be the fact that middle school students were younger than high school students, and younger adolescents have closer proximity between the case and their peers than older adolescents [40], and there might be more progression rate from infection to active TB at young age. In Guangxi, non-Han-ethnicity people were more likely to come from impoverished mountain areas, with limited resources for disease prevention and control, and thus had contributors to the burden of TB disease.

Our study was limited by the small number of isolates from active TB cases; spoligotyping and MIRU-VNTR genotyping could not classify strains into specific sublineages. Therefore, the explanation for genotypes of index cases and contact screening outcome may be incomplete.

## 5. Conclusion

The Beijing strains of index cases were not independently associated with increased TB infection/being active TB among contacts compared to the non-Beijing strains. The contribution of characteristic factors to the contact screening outcome should be taken into account for school contact investigations.

## Figures and Tables

**Table 1:** Characteristics of index cases by genotypes of *M.tb* (N=117).

Variable	Genotype of index case [n (%)]			*P-value (Chi-squared Test)*
	Beijing (n=7)	Non-Beijing (n=9)	Unknown (n=101)	
*School type*				
Middle school	3 (6.7)	2 (4.4)	40 (88.9)	0.701
High school	4 (5.6)	7 (9.7)	61 (84.7)	
*Sex*				
Male	0 (0)	8 (14.5)	47 (85.5)	**0.001**
Female	7 (11.3)	1 (1.6)	54 (87.1)	
*Ethnicity*				
Han	5 (7.9)	5 (7.9)	53 (84.1)	0.666
Others	2 (3.7)	4 (7.4)	48 (88.9)	
*BCG scar*				
Absent	3 (7)	4 (9.3)	36 (83.7)	0.768
Present	4 (5.4)	5 (6.8)	65 (87.8)	
*Sputum smear status*				
Negative	5 (6.8)	5 (6.8)	64 (86.5)	0.839
Positive	2 (4.7)	4 (9.3)	37 (86)	
*Cavity on chest*				
No	4 (4.8)	6 (7.2)	73 (88)	0.61
Yes	3 (8.8)	3 (8.8)	28 (82.4)	

**Table 2:** Characteristics of contacts (N=6512) and contact screening outcomes by genotypes of index cases.

Variable	Genotype of index case [n (%)]			*P-value *(Chi-squared Test)
	Unknown (n=5625)	Non-Beijing (n=468)	Beijing (n=419)	
*School type*				
Middle school	2156 (88.6)	82 (3.4)	196 (8.1)	< 0.001
High school	3469 (85.1)	386 (9.5)	223 (5.5)	
*Sex*				
Male	2788 (85.6)	231 (7.1)	237 (7.3)	0.021
Female	2837 (87.1)	237 (7.3)	182 (5.6)	
*Ethnicity*				
Han	2339 (92.5)	164 (6.5)	26 (1.0)	< 0.001
Others	3286 (82.5)	304 (7.6)	393 (9.9)	
*BCG scar*				
Absence	3910 (85.1)	384 (8.4)	302 (6.6)	< 0.001
Presence	1715 (89.5)	84 (4.4)	117 (6.1)	
*Screening results*				
0-5 mm	4623 (86.8)	407 (7.6)	298 (5.6)	< 0.001
6-9 mm	544 (83.8)	26 (4)	79 (12.2)	
10-14 mm	238 (84.4)	18 (6.4)	26 (9.2)	
≥ 15 mm (without TB)	183 (88)	10 (4.8)	15 (7.2)	
≥ 15 mm (with TB)	37 (82.2)	7 (15.6)	1 (2.2)	

**Table 3:** Multivariate ordered logistic regression analysis to choose the best model.

Variables	Model 1	Model 2	Model 3	Model 4	Model 5	Model 6
Index case genotypes	+	+	+	+	+	+
Sex of index case	-	+	+	+	+	+
School types	-	-	+	+	+	+
Sex of contacts	-	-	-	+	+	+
Ethnicity of contacts	-	-	-	-	+	+
BCG scar of contacts	-	-	-	-	-	+
AIC	8762.1	8743.9	8692.0	8692.4	8686.7	8631.5
*P-value* (Likelihood ratio test)		<0.001^c^	<0.001 ^c^	0.200 ^c^	0.010 ^d^	<0.001^d^

Note:  ^c^compared to the preceding model;  ^d^compared to Model 3.

**Table 4:** Ordered logistic regression for genotypes of index cases to the outcome of contact screening.

Variables	Contact screening results					Ordinal adjusted OR (95% CI)	*P-value*
	0-5 mm	6-9 mm	10-14 mm	≥ 15 mm (without TB)	≥ 15 mm (with TB)		
*Index case genotypes*							
Non-Beijing	407 (87)	26 (5.6)	18 (3.8)	10 (2.1)	7 (1.5)	1	
Beijing	298 (71.1)	79 (18.9)	26 (6.2)	15 (3.6)	1 (0.2)	1.34 (0.93, 1.95)	0.057
Unknown	4623 (82.2)	544 (9.7)	238 (4.2)	183 (3.3)	37 (0.7)	1.12 (0.85, 1.51)	0.221
*Sex of index case*							
Male	2575 (84.8)	291 (9.6)	82 (2.7)	64 (2.1)	26 (0.9)		
Female	2753 (79.2)	358 (10.3)	200 (5.8)	144 (4.1)	19 (0.5)	1.39 (1.21, 1.60)	<0.001
*School type*							
High school	3453 (84.7)	383 (9.4)	121 (3)	100 (2.5)	21 (0.5)	1	
Middle school	1875 (77)	266 (10.9)	161 (6.6)	108 (4.4)	24 (1)	1.65 (1.45, 1.88)	<0.001
*Sex of contacts*							
Male	2671 (82)	348 (10.7)	140 (4.3)	75 (2.3)	22 (0.7)	1	
Female	2657 (81.6)	301 (9.2)	142 (4.4)	133 (4.1)	23 (0.7)	1.10 (0.97, 1.25)	0.07
*Ethnicity of contacts*							
Han	2196 (86.8)	128 (5.1)	102 (4)	69 (2.7)	34 (1.3)	1	<0.001
Non-Han	3132 (78.6)	521 (13.1)	180 (4.5)	139 (3.5)	11 (0.3)	1.71 (1.48, 1.97)	
*BCG scar of contacts*							
Present	3810 (82.9)	397 (8.6)	211 (4.6)	143 (3.1)	35 (0.8)	1	
Absent	1518 (79.2)	252 (13.2)	71 (3.7)	65 (3.4)	10 (0.5)	1.19 (1.04, 1.37)	<0.001

## Data Availability

The [.xlsx] data used to support the findings of this study are included within the supplementary information files (available here).

## Supplementary Materials

Supplementary Materials (1) Supplementary materials file “Index case.xlsx” is the dataset for Table 1 of the manuscript, “Characteristics of index cases by genotypes of M.tb (N=117)”. (2) Supplementary materials file “Contact.xlsx” is the dataset for Table 2 and Figures 1, 4(a), and 4(b) of the manuscript. (3) Supplementary materials file “Genotype.xlsx” is the dataset for Figures 2 and 3 of the manuscript.
